# Achieving Food System Transformation: Insights From A Retrospective Review of Nutrition Policy (In)Action in High-Income Countries

**DOI:** 10.34172/ijhpm.2020.188

**Published:** 2020-10-19

**Authors:** Amanda J. Lee, Katherine Cullerton, Lisa-Maree Herron

**Affiliations:** School of Public Health, Faculty of Medicine, The University of Queensland, Brisbane, QLD, Australia.

**Keywords:** Nutrition Policy, Food Systems, Sustainability, Equity, NOURISHING Framework, OECD countries

## Abstract

**Background:** Comprehensive nutrition policies are required urgently to help transform food systems to more equitably deliver healthy, sustainable diets.

**Methods:** Literature was searched systematically for nutrition policies of the then 34 Organisation for Economic Co-operation and Development (OECD) members as part of a scoping study. Recently, results were re-analysed, against the NOURISHING framework.

**Results:** Twenty-three nutrition policy documents were identified for 19 jurisdictions. Most policy actions focused on the behaviour change communication domain: all (100%) promoted consumption of ‘healthy’ choices. In the food environment domain, most policies included food labelling (84%), product reformulation (68%), providing healthy foods in public institutions (89%, mainly schools), and restricting food advertising (53%), largely through voluntary codes. Relatively few economic tools were being applied. There was very little focus on reducing consumption of ‘unhealthy’ food or drinks. Not all nutrition policy actions identified were covered by the NOURISHING framework.

**Conclusion:** The NOURISHING framework could be expanded to more comprehensively encompass the health and sustainability dimensions of food systems, eg, by detailing optimum governance arrangements. As recently as seven years ago, half of the most developed economies globally did not have a publicly available nutrition policy. Existing policies were dominated by conventional nutrition education approaches, while policy actions targeting food environments, and regulatory and legislative reforms, were rare. This is consistent with a neo-liberal approach centring individual responsibility. No examples of the multi-strategy, inter-sectoral, coordinated, evidence-based policies required to drive systemic transformation were identified. Therefore, it is not surprising that rates of obesity and diet-related conditions have continued to rise in these jurisdictions, nor that governments are currently off-track to deliver the systemic transformation required to meet relevant global health and sustainable development goals.

## Introduction

There is an urgent need globally for transformation of food systems to more equitably deliver healthy, sustainable diets.^1-4^ Achieving the necessary systemic change requires a multisectoral response with strong governance and accountability to mobilise action by all governments, and other sectors.^1,5^ Food system approaches holistically consider aspects of both supply and demand, encompassing food supply chains from producers to consumers; the food environment that drives consumer choice, and knowledge, attitudes and behaviors.^2,3^ However such approaches can be very challenging for countries to realise due to the heterogeneous nature of stakeholders relevant to nutrition who often have different (or conflicting) worldviews, vested interests and different levels of power and influence.^6,7^ A range of nutrition policy actions can be applied to address systemic leverage points at country level.^8,9^ Yet, many national nutrition policies continue to perpetuate conventional perspectives.^5^ Applying the lens of political economy highlights how policy processes are shaped by power, incentives, institutions and ideas.^10^ Better understanding of the scope, content and evolution of nutrition policy actions can help inform this approach. A number of food system and policy frameworks, of different scope, have been developed to classify these potential nutrition policy actions.^5,11^

A scoping study to inform development of a new nutrition policy in Australia in 2013^12^ provided the opportunity to identify and review nutrition policies of high-income countries (members of the Organisation for Economic Co-operation and Development [OECD]). These countries were considered most likely to have capacity and resources to develop and implement evidence-based nutrition policies that comprehensively addressed key leverage points in food systems. This review recently re-analysed the results of one component of that study – the examination of the development process and content of existing international nutrition policies – against a high profile policy framework developed subsequently, the World Cancer Research Fund’s NOURISHING framework.^11,13^ The framework was designed to help policy-makers, researchers and community organisations identify and advocate for key food policy interventions needed to develop a “comprehensive policy package” to promote healthy diets.^14^

 The aim of this systematic scoping review was to classify nutrition actions included in the identified policies to assess activity. A secondary aim was to determine whether any identified nutrition policy actions were not encompassed by the NOURISHING framework, to highlight potential actions to tackle food systems more broadly.

The NOURISHING framework has been used to categorise ‘best practice’ policy actions^14^ and report on progress and recommendations around key policy actions.^15^ However, to our knowledge this is the first time it has been used cross-sectionally as a tool to assess and quantify the scope and breadth of policy actions included in real world nutrition policies of a group of countries.

## Methods

This review recently re-analysed the results of a systematic literature review that was conducted as one component of a scoping study to inform development of a new nutrition policy for Australia in 2013.^16,17^ A new Australian nutrition policy was not developed subsequently. However, the scoping study was released in full in March 2016 following a request under the Freedom of Information Act 1982^18^; the full report is available online, including data assessed in this study (in Section 3.2 and Appendix 6 of the report).^16^ This provided an opportunity to re-analyse the data to ‘benchmark’ policy action as at 2013, to provide insights into the breadth and scope of nutrition policy actions implemented by governments, and to support monitoring and assessment of subsequent nutrition-related policy action. Re-analysis, as described below, was completed in late 2019.^16^

The original multi-component scoping study was conducted using standard methods for a scoping review conducted in a systematic manner^19,20^ and in accordance with the Preferred Reporting Items for Systematic Reviews and Meta-Analyses (PRISMA) Statement.^21^ For this review, the study population was limited to the then 34 member nations of the OECD, as these nations had similar socio-political and economic systems as Australia, and were considered likely to have sufficient capacity and resources to develop and implement comprehensive nutrition-related policies. The scope was limited to national or regional nutrition-related policy or strategy documents that included policy actions aimed at improving nutrition and/or reducing the incidence and prevalence of diet-related risk factors and diseases (including obesity). Policies needed to be publicly accessible (ie, retrievable via website or literature search) as this was considered necessary for potentially effective implementation. Nutrition policy actions were defined as discrete elements of broader policies that aimed to improve dietary intake and/or food environments. The period of inclusion was 2002 to 2013. Included documents were those available in English. The detailed search strategy is provided as Supplementary file 1. The answerable question followed the PICO-T concept:

- Population: In scope countries included Australia; Austria; Belgium; Canada; Chile; Czech Republic; Denmark; Estonia; Finland; France; Germany; Greece; Hungary; Iceland; Ireland; Israel; Italy; Japan; Korea; Luxembourg; Mexico; the Netherlands; New Zealand; Norway; Poland; Portugal; Slovak Republic; Slovenia; Spain; Sweden; Switzerland; Turkey; the United Kingdom (England, Scotland and Northern Ireland); and the United States.
- Intervention: Existence of national nutrition-related policies in these countries, targeting the whole population or sub-groups. Excluded were policies targeting those with serious medical conditions requiring specialised dietary advice, elite athletes and/or frail elderly in institutions.
- Comparator: Lack of publicly available nutrition policy.
- Outcome: Existence of nutrition policy actions in relevant policies or strategies. Search terms included “diet*” “nutr*” “food” “bev*” “polic*” “strat*” “progr*” “plan” “project” “prevent*” “intervention” “initiat*” “obes*” “non-communicable dis*” “guide*” “reg*” “legislat*” “law” “direct*” “environm*” “food supply”
- Type of study: Food and Nutrition: policies, policy, strategy, plan, report, monitoring, surveillance, guideline, guidance, legislation, program, project, intervention, initiative, regulation, law, directive, evaluation.

The choice of electronic databases extended beyond those focused on health to include relevant areas such as agriculture, environment and transport, including the Cochrane Public Health Group Specialised Register, the Cochrane Library, MEDLINE, MEDLINE In-Process, EMBASE, CINAHL, ASSIA, EPPI Centre, DoPHER, TRoPHI, ERIC, Sociological Abstracts, Transport Database TRIS, Web of Science: Science Citation Index, Social Sciences Citation Index and Conference Proceedings Citation Index, Agris, HEED and NEED. Websites searched included the European Union (EU) Platform on Diet, Physical Activity and Health^22^; Health Evidence^23^; International Union for Health Promotion and Education^24^; Health Technology and Assessment Programme^25^; National Institute for Health and Care Excellence^26^; Scottish Intercollegiate Guidelines Network^27^; US Centres for Disease Control and Prevention^28^; World Health Organization (WHO)^29^; WHO global database on the implementation of nutrition action^30^; the Food and Agriculture Organization of the United Nations^31^; and government websites of each of the then 34 the OECD member nations, including health, education, community service, agriculture, and environment agencies. Websites were searched with the entry of each search term in the Google search engine, and the first 10 pages of returns were reviewed to identify any references to nutrition policy.

Data analysis involved extraction of the scope, format, evidence base, coordination mechanisms and content (policy issues and actions) of identified national and regional policy documents, as described in detail in the scoping study.^16^ Although all included nutrition policies were current, the date of implementation of policy actions was not always clear.

 This review mapped extracted nutrition policy actions against the NOURISHING framework, effectively re-synthesising the results of the scoping study to answer the research question: “What were the scope and key inclusions (nutrition policy actions) of national nutrition policies of OECD countries in 2013?”

**Figure 1:**
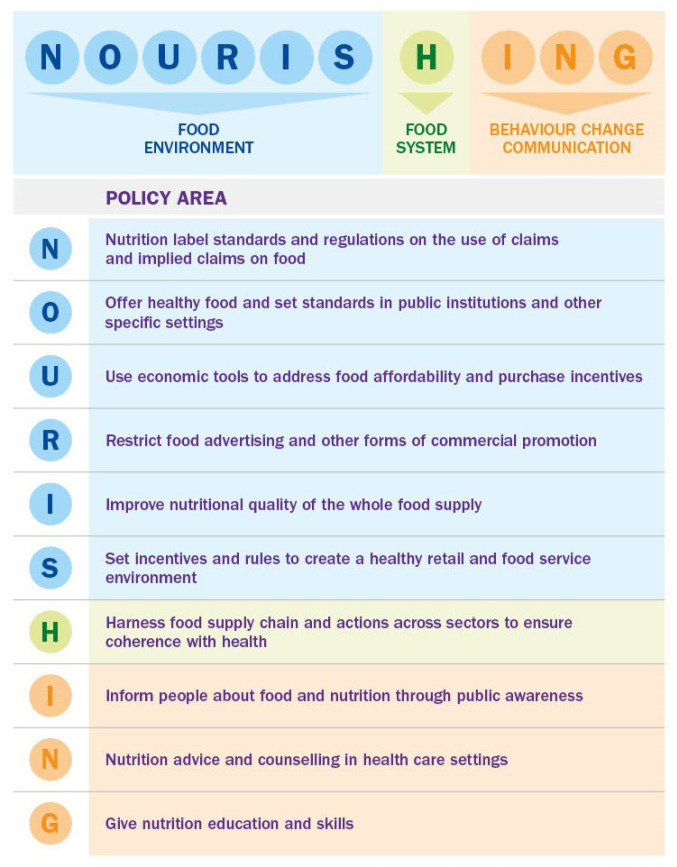
The NOURISHING Policy Framework to Promote Healthy Diets and Reduce Obesity.^13^ Reproduced with permission of the World Cancer Research Fund (WCRF).

The NOURISHING framework covers 10 policy areas across three domains which are presented as collectively forming a “comprehensive” nutrition policy package.^14^ Each letter in the word NOURISHING represents an area where policy action is required (Figure 1).

 National nutrition policies that met the inclusion criteria were scrutinised line by line for terms corresponding to each of the 10 policy action areas in the framework; these were captured and the results were tabulated. Each stage of the search, data extraction and mapping process was conducted by one researcher, with 10% of abstracts, extractions and synthesis cross checked by AL, to help control for observer bias.

 The EU and US policies were higher level strategy instruments than most national nutrition policies, and there was also marked variation in the span and depth of different national policies, hence it was not always possible to identify the detail of specific nutrition policy actions required to categorise them confidently against the NOURISHING framework. Included policy actions that did not correspond to any of the 10 action areas in the framework were noted and classified separately.

The intent was to capture the breadth and scope of each national nutrition policy. Included policies were assessed for specific nutrition policy action; statements such as “investigate the feasibility of …” were not considered evidence of policy intent. In relevant areas, policy actions were sub-classified according to types of examples provided in the NOURISHING framework^13^ and whether they aimed to promote ‘healthy’ foods or restrict ‘unhealthy’ foods, as defined by each nation’s relevant food-based dietary guidelines, where available.

## Results

**Figure 2:**
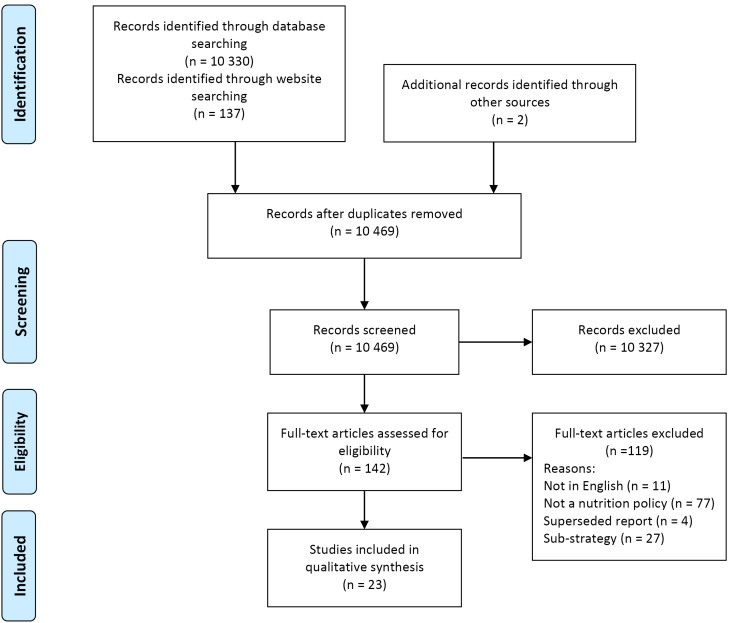
PRISMA Flow Diagram. Abbreviation: PRISMA, Preferred Reporting Items for Systematic Reviews and Meta-Analyses

The PRISMA diagram presenting the search and screening process is included at Figure 2.

A total of 23 health and nutrition policies were identified, for 17 of the OECD member countries and two regions (n = 19 jurisdictions). The jurisdictions with included nutrition policies, and the titles and web addresses (URL) of the documents, are listed in Table 1. The policies of some countries, for example Finland, were identified in regional policies, but not as separate countries. Within the time period searched, multiple policies were found for Denmark, France, the United States and the EU; all documents were included for description of scope (n = 23), but for the analysis of the content of nutrition policy actions, only the most recent policies were included (n = 19). All nutrition policies were identified in the database and website searches, with the exception of Canada’s, which was identified through hand searching of references.

**Table 1:** Included National Nutrition Policies

**Jurisdiction**	**Policy Document Title and Year of Publication**	**URL** ^a^	**Source** ^*^
Australia	Food and Nutrition Policy (1992)^32^	https://extranet.who.int/nutrition/gina/sites/default/files/AUS%201992%20Food%20and%20nutrition%20policy%20.pdf	W
Belgium	National Food and Health Plan for Belgium 2005-2010 (2005)^33^	http://www.health.belgium.be/internet2Prd/groups/public/@public/@dg4/@consumerproducts/documents/ie2divers/7526403.pdf ^a^	W
Canada	The Integrated Pan-Canadian Healthy Living Strategy 2005 (2005)^34^	http://www.phac-aspc.gc.ca/hp-ps/hl-mvs/ipchls-spimmvs/index-eng.php	H
Czech Republic	Food Safety and Nutrition Strategy 2010-2013 (2010)^35^	http://eagri.cz/public/web/file/44930/Strategie_BP_EN.pdf	W
Denmark	Healthy Throughout Life – the Targets and Strategies for Public Health Policy of the Government of Denmark, 2002–2010 (2003)^36^ National Action Plan Against Obesity: Recommendations and Perspectives (Short Version) (2003)^37^	http://dopah.anamai.moph.go.th/upload/fckeditor/file/PA_PLAN_5.pdf ^a^https://extranet.who.int/nutrition/gina/en/node/8443	W W
England	Healthy Weight, Healthy Lives: A Cross-Government Strategy for England, 2008 (2008)^38^	http://webarchive.nationalarchives.gov.uk/20100407220245/http://www.dh.gov.uk/prod_consum_dh/groups/dh_digitalassets/documents/digitalasset/dh_084024.pdf	W
European Union	First action Plan for Food and Nutrition Policy 2000-2005, WHO European Union (2001)^54^ WHO European Action Plan for Food and Nutrition Policy 2007-2012 (2008)^55^	http://www.euro.who.int/__data/assets/pdf_file/0013/120244/E72199.pdf http://www.euro.who.int/__data/assets/pdf_file/0017/74402/E91153.pdf	W W
France	Programme National Nutrition Santé, 2001-2005 [National Nutrition Health Programme] (2001)^39^ Deuxième Programme National Nutrition Santé‚ 2006-2010 (2006) [Second National Nutritional Health Programme]^40^	https://extranet.who.int/nutrition/gina/en/node/8515 https://extranet.who.int/nutrition/gina/en/node/8102	D W
Hungary	‘Johan Béla’ National Programme for the Decade of Health, 2004 (2004)^41^	http://ec.europa.eu/health/archive/ph_determinants/socio_economics/documents/hungary_rd01_en.pdf	W
Ireland	Obesity: The Policy Challenges – the Report of the National Taskforce on Obesity 2005 (2005) ^42^	https://www.hse.ie/eng/health/child/healthyeating/taskforceonobesity.pdf	W
Israel	Health Behaviors: Promoting Physical Activity, Prevention and Treatment of Obesity, Healthful Nutrition (Healthy Israel 2020) (2011)^43^	https://extranet.who.int/nutrition/gina/sites/default/files/Health%20Behaviors_MOH_Israel.pdf	W
Italy	Guadagnare Salute Italia 2007 [Gaining Health Action Plan: Encouraging Healthy Choices] (2007)^44^	http://www.salute.gov.it/imgs/C_17_pubblicazioni_605_allegato.pdf	W
Japan	Basic Plan for Promotion of Shokuiku (Food Education), 2005 (2005)^45^	http://www.maff.go.jp/e/pdf/shokuiku.pdf	W
New Zealand	Healthy Eating – Healthy Action: Oranga Kai – Oranga Pumau: Strategy Framework (HEHA Strategy) (2003)^46^ Healthy Eating – Healthy Action: Oranga Kai – Oranga Pumau: Implementation Plan (2004-2010) (2003)^47^	http://www.health.govt.nz/publication/healthy-eating-healthy-action-oranga-kai-oranga-pumau-strategic-framework http://www.health.govt.nz/publication/healthy-eating-healthy-action-oranga-kai-oranga-pumau-implementation-plan-2004-2010	D W
Nordic Region/ Scandinavia	Health, Food and Physical Activity: Nordic Plan of Action on Better Health and Quality of Life Through Diet and Physical Activity (2006)^53^	https://www.norden.org/en/publication/health-food-and-physical-activity	W
Scotland	Healthy Eating, Active Living: An Action Plan to Improve Diet, Increase Physical Activity and Tackle Obesity, Scotland (2008-2011) (2008)^48^	http://www.scotland.gov.uk/Publications/2008/06/20155902/0	W
Spain	Estrategia NAOS: Spanish Strategy for Nutrition, Physical Activity and Prevention of Obesity (2005)^49^	http://www.aecosan.msssi.gob.es/AECOSAN/docs/documentos/nutricion/NAOS_Strategy.pdf	D
Switzerland	Swiss Nutrition Policy 2013–2016: Based on the Main Findings of the 6Th Swiss Nutrition Report (2012)^50^	https://extranet.who.int/nutrition/gina/sites/default/files/CHE%202013-2016%20Swiss%20Nutrition%20Policy%20EN.pdf	W
The United States	Healthy People 2020: Nutrition and Weight Status (2010)^51^ National Prevention Strategy: America’s Plan for Better Health and Wellness (2011)^52^	http://www.healthypeople.gov/2020/topicsobjectives2020/overview.aspx?topicid=29 https://www.hhs.gov/sites/default/files/disease-prevention-wellness-report.pdf	W D

Abbreviations: WHO, World Health Organization; NAOS, Strategy for Nutrition, Physical Activity and Prevention of Obesity. ^a^ These policy documents were no longer located at these URLs as in 2013 and could not be located elsewhere. * D = document identified through database searches (see Methods), W = document identified through website searches, H = identified through hand search of reference lists.

###  Policy Scope and Focus

There was a wide range of approaches to national nutrition policies (Tables 1, 2 and 3). Of all 23 policy documents, seven were ‘stand-alone’ nutrition policies, while 10 combined nutrition with physical activity specifically in policies to address obesity. The United States, Canada and Italy also included other risk factors, such as cigarette smoking and alcohol intake, in broader policies to prevent non-communicable diseases (NCDs). The three regional documents focused on nutrition alone, and were relatively comprehensive. Policies targeting obesity as well as nutrition tended to be more recent; however some of these provided less detail about dietary components. For example, Canada’s strategy had a strong focus on physical activity and sedentary behaviour, with the nutrition elements focussed mainly on increasing fruit and vegetable consumption. Italy, alone, adopted a multiple risk factor approach while maintaining a strong focus on nutrition. The most comprehensive dedicated nutrition policies came from England, France, Israel, Scotland, Ireland, Hungary and some Nordic countries, particularly Denmark. The policies of countries recognised as having a strong food-based culture, notably France and Italy, were more likely to be focussed on foods rather than nutrients; others tended to adopt a more nutrient-based approach.

**Table 2:** Policy Actions in Included Nutrition Policy Documents

	**NOURISHING Framework Policy Area **	**Examples**	**Healthy/** **Unhealthy Foods**	**AU **	**BE**	**CA**	**CZ**	**DK (2)**	**ENG**	**FR (2)**	**HU**	**IE**	**IL**	**IT**	**JP**	**NZ**	**SCT**	**ES**	**CH**	**US (2)**	**NR**	**EU **	**Tally (n) of 19 Jurisdictions**	**% Of Jurisdictions **
**Food Environment**																								
**N**	Nutrition label standards and regulations on the use of claims and implied claims on foods	Nutrient lists on food packages; clearly visible ‘interpretive’ and calorie labels; menu, shelf labels; rules on nutrient and health claims	Nutrient-focused Nutrient and kJ content, claims and health claims	√		√	√	√	√	√	√	√	√	√	√	√		√			√	√	16	84
			Nutrient-focused ‘Interpretive' labels on overall food quality					√	√				√								√		4	21
**O**	Offer healthy foods and set standards in public institutions and other specific settings	Fruit and vegetables programmes; standards in education, work, health facilities; award schemes; choice architecture	Healthy foods and drinks	√		√	√	√	√	√	√	√	√	√	√	√	√	√		√	√		17	89
			Unhealthy foods and drinks						√	√			√	√			√						5	26
**U**	Use economic tools to address food affordability and purchase incentives	Targeted subsidies; price promotions at point of sale; unit pricing; health-related food taxes	Healthy foods and drinks			√		√	√			√	√				√			√			7	37
			Unhealthy foods and drinks					√		√	√		√								√		5	26
**R**	Restrict food advertising and other forms of commercial promotion	Restrict advertising to children that promotes unhealthy diets in all forms of media; sales promotions; packaging; sponsorship	Targets unhealthy foods and drinks		√	√		√	√				√	√			√	√	√		√		10	53
**I**	Improve the nutritional quality of the whole food supply	Reformulation to reduce salt and fats; elimination of trans fats; reduce energy density of processed foods; portion size limits	Addresses both increase of healthy options and decrease of unhealthy options	√		√	√	√	√		√		√	√		√	√	√		√		√	13	68
**S**	Set incentives and rules to create a healthy retail and food service environment	Incentives for shops to locate in underserved areas; planning restrictions on food outlets; in-store promotions	Promotes healthy foods and drinks		√				√	√										√			4	21
			Targets unhealthy foods and drinks																				0	0
**Food System**																								
**H**	Harness the food supply chain and actions across sectors to ensure coherence with health	Supply-chain incentives for production; public procurement through ‘short’ chains; health-in-all policies; governance structures	Considers both healthy and unhealthy options within a food systems approach						√ Local level	√ Local level +		√		√ Local level +		√ Local level	√ Local level	√ Local level		√	√	√	10	53
**Behaviour Change Communication**																								
**I**	Inform people about food and nutrition through public awareness	Education about food-based dietary guidelines, mass media, social marketing; community and public information campaigns	Focus on healthy foods	√		√	√	√	√	√	√	√	√	√	√	√	√	√	√	√	√	√	19	100
			Focus on unhealthy foods					√	√	√				√			√				√		6	32
**N**	Nutrition advice and counselling in healthcare settings	eg, Nutrition advice for at-risk individuals; telephone advice and support; clinical guidelines for health professionals on effective interventions for nutrition	Targets both healthy and unhealthy foods	√	√	√	√	√	√	√	√	√	√	√	√	√	√	√	√	√	√	√	19	100
**G**	Give nutrition education and skills	Nutrition, cooking/food production skills on education curricula; workplace health schemes; health literacy programs	Healthy foods	√	√	√	√	√	√	√	√	√	√	√	√	√	√	√	√	√	√	√	19	100
			Unhealthy foods										√								√		2	11
Nutrition policy actions to promote healthy food (% of possible 16 policy action areas)				6 (38)	7 (44)	8 (50)	6 (38)	11 (69)	13 (81)	10 (63)	7 (44)	7 (44)	12 (75)	9 (56)	5 (31)	7 (44)	9 (56)	6 (38)	4 (25)	8 (50)	11 (69)	6 (38)		
Additional elements as detailed in Table 3 (n)				11	13	11	8	9	18	20	9	6	8	7	5	14	9	6	10	13	12	12		
Total (% of potential 44)				17 (39)	20 (45)	19 (43)	14 (32)	20 (45)	31 (70)	30 (68)	16 (36)	13 (30)	20 (45)	16 (36)	10 (23)	21 (48)	18 (41)	12 (27)	14 (32)	21 (48)	23 (52)	18 (41)		

Abbreviations: AU, Australia; BE, Belgium; CA, Canada; CZ, Czech Republic; DK, Denmark; ENG, England; FR, France; HU, Hungary; IE, Ireland; IL, Israel; IT, Italy; JP, Japan; NZ, New Zealand; SCT, Scotland; ES, Spain; CH, Switzerland; NR, Nordic Region; EU, European Union; US, United States. + Multiple actions at local level

**Table 3:** Elements of Included Policies Not Captured in the NOURISHING Framework

	**Examples**	**AU **	**BE**	**CA**	**CZ**	**DK (2)**	**ENG**	**FR (2)**	**HU**	**IE**	**IL**	**IT**	**JP**	**NZ**	**SCT**	**ES**	**CH**	**US (2)**	**NR**	**EU**	**Tally (n) of 19 Jurisdictions**	**% Of Jurisdictions**
**Foundation Tools**																						
Development of food-based evidence-informed national dietary guidelines		√	√	√	√	√	√	√	√			√	√	√		√	√	√	√	√	16	84
Population monitoring and surveillance systems	Diet-related disease	√	√	√	√	√	√	√	√	√	√	√	√	√	√	√	√	√	√		18	95
	Dietary intake		√	√	√	√	√	√	√	√	√	√	√	√	√	√	√	√	√		17	89
	Nutrition knowledge and attitudes		√					√									√	√			4	21
	Food supply													√							3	16
	Food expenditure																				1	5
	Food environments																				0	0
**Governance Mechanisms (Process and Structural Factors)**																						
Coordination mechanism and governance		√	√	√	√		√		√	√	√	√		√		√		√	√	√	15	79
Clear, comprehensive goals and targets			√			√		√	√		√							√	√	√	8	42
Dedicated funding							√	√							√						3	16
Supports implementation research		√	√	√	√		√	√										√	√		9	47
Demonstration programs		√			√		√														3	16
Policy evaluation		√		√		√	√	√	√	√				√		√		√	√	√	12	63
**Specific Policy Actions/Evidence-Based Interventions**																						
Environmental sustainability								√						√			√				4	21
Food safety					√								√					√		√	5	26
Trade																					1	5
Body dysmorphia/eating disorders						√		√													2	10
Undernutrition/nutrient deficiencies			√			√									√			√		√	7	37
Breastfeeding		√	√				√	√							√				√	√	7	37
**Specific Focus Areas**																						
Equity		√	√	√	√	√	√	√	√	√	√			√	√			√			13	68
Indigenous/First Nations peoples		√		√										√							3	16
Supportive nutrition environments			√	√			√	√	√		√				√		√		√		10	53
Obesity		√				√	√							√			√		√	√	7	37
Physical activity			√	√			√	√		√		√		√	√	√	√	√			11	58
Economic benefits																			√	√	2	10
Fruit and vegetables				√			√	√							√						5	26
Salt		√	√				√	√			√	√		√			√		√	√	10	53
Alcohol												√	√	√				√			5	26

Abbreviations: AU, Australia; BE, Belgium; CA, Canada; CZ, Czech Republic; DK, Denmark; ENG, England; FR, France; HU, Hungary; IE, Ireland; IL, Israel; IT, Italy; JP, Japan; NZ, New Zealand; SCT, Scotland; ES, Spain; CH, Switzerland; NR, Nordic Region; EU, European Union; US, United States.

 Most policies included nutrition strategies targeted at children (n = 18, 78%) and infants (n = 13, 56%) – mainly through breastfeeding promotion; about half targeted pregnant and lactating women (n = 11, 48%) and those in lower socioeconomic groups (n = 10, 43%). Few countries (less than 20%), including England, Ireland and the United States, included specific strategies to improve diets of people in lower income groups, such as through social welfare programs. Denmark and France were the only countries that identified older people as a focus. References to Indigenous/First Nations Peoples were included only in the policies of three countries.

###  Policy Content 

**Figure 3:**
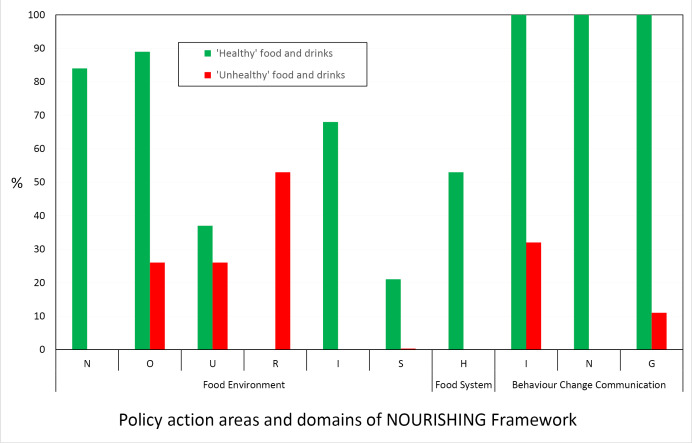
Proportion of Country/Regional Nutrition Policies Including Actions in Each NOURISHING Framework Area.

The specific nutrition policy actions described in the 19 included jurisdictional nutrition policies are mapped against the NOURISHING framework’s three domains and 10 policy action areas in Table 2 and summarised in Figure 3. Policy actions that did not correspond readily to areas in the NOURISHING framework are presented in Table 3. Detailed descriptions of each jurisdiction’s policy actions are available elsewhere (Section 3.2 and Appendix 6 of the scoping study).^16^

###  Policy Actions in the Food Environment Domain

The ‘Food Environment’ domain of the NOURISHING framework includes 6 of the 10 policy areas (‘NOURIS’) (Figure 1, Table 2).

####  Nutrition Label Standards and Regulations on Use of Claims on Foods (N)

 The majority of included nutrition policies (84%) contained actions related to food labelling, with the most common initiatives being mandating nutrient lists on food packaging and front-of-packaging labelling. Several policy documents identified the need for mandatory, simplified ‘interpretive’ front-of-pack labelling systems to support more informed food choices; this was emphasised in the policies of England, Hungary and France where ‘traffic light labelling’ based on nutrient profiling was favoured. Several more countries were at an exploratory stage, reporting intention or research underway to identify an appropriate interpretive labelling model to help consumers recognise nutritionally healthy or unhealthy foods. No country or region included, or identified the need for, specific warning labels on unhealthy food and drinks in nutrition policy documents.

 A related expressed concern was the need for verification and regulation of health claims on food packaging (eg, in Belgium, France and Spain), which were seen as having potential to undermine evidence-based dietary guidelines and nutrition messages. The Irish policy also committed to “rigorous and regular review” of all products that claimed to support weight loss.

 The need for more transparent nutrition information at point of sale, specifically menu labelling in restaurants including on menu boards in quick service restaurants, was identified in three included policies (the United States, Spain and Israel).

####  Offer Healthy Foods and Set Standards in Public Institutions and Other Specific Settings (O)

 In most policy documents (89%) there was a focus on increasing the availability of healthy foods in public institutions, mainly schools (74%) and child care centres (13%). However, only about one-third (35%) of countries addressed food supply in other settings, most commonly public healthcare settings, including hospitals (26%). Policy actions predominantly targeted increasing supply of fruit and vegetables and dairy foods, for example the provision of free fruit and vegetables and milk in schools in some EU countries since 2009, and the school milk scheme in Nordic countries. Some countries (eg, New Zealand) reported supplying fruit to schools in low socioeconomic areas. Several provided healthy cooked school lunches. Health promotion settings approaches, including implementation of nutritional policies/guidance and accreditation, were in place in child care centres in France, Belgium, Hungary, and Israel. Several countries/regions had also introduced award schemes to recognise achievements in improving food supply.

 Far fewer countries had implemented actions aimed at decreasing the supply of unhealthy foods and drinks (26%). France and the United Kingdom had banned vending machines in schools, and Israel had banned ‘unhealthy’ foods in schools. Italy targeted supply of ‘fast foods’ and energy-dense, nutrient-poor products in vending machines in healthcare settings. Beyond these initiatives in school and healthcare settings, there were no strategies aimed at restricting supply of unhealthy foods and drinks in other settings in any country.

 While the need to address food supply issues was identified frequently, specific initiatives, roles and responsibilities for action were detailed rarely. In particular, activities to decrease supply of unhealthy food and drinks tended to be articulated in formative language, such as in statements of intent “to investigate” or “to develop” approaches, rather than to actually implement initiatives.

####  Use Economic Tools to Address Food Affordability and Purchase Incentives – Targeted Subsidies; Price Promotions at Point of Sale; Unit Pricing; Health-Related Food Taxes (U)

 Economic tools to address food affordability and encourage healthier diets, such as differential taxation, agricultural subsidies and targeted support for socioeconomically disadvantaged population groups, were included in 10 policy documents (53%). Whole of population policy tools were used by only a few jurisdictions to promote healthy choices (16%) or discourage unhealthy choices (11%). Others acknowledged the need for some form of fiscal action to address comparative affordability of ‘healthy’ and ‘unhealthy’ foods, but outlined work that was largely formative. For example, Canada’s policy stated that potential fiscal policy responses such as subsidies for healthy food would “be investigated,” and Ireland planned research into the influence of fiscal policies on consumers’ food purchasing. However, Ireland’s included an action to review social welfare (assistance) payments also.

 The descriptions of differential taxation suggest a primary purpose of revenue-raising rather than improving health outcomes. Of the countries applying differential taxation systems specifically for stated reasons of nutrition promotion, Denmark had introduced a ‘fat tax’ (subsequently revoked), Hungary had a ‘junk food tax,’ France taxed sugar-sweetened beverages and Israel taxed unhealthy foods “such as soda and/or trans fats.” Denmark, Norway, Iceland and Finland also reported higher taxation rates on sugar, chocolate and sugar sweetened beverages than on healthy foods and drinks.

 In Canada, France and the United Kingdom, differential application of a goods and services tax (GST) or value-added tax on some foods occurred, although these broader fiscal policies were not necessarily identified as nutrition initiatives in the documents reviewed. In the United Kingdom, food was value-added tax zero-rated except for food items provided as catering, takeaways or in restaurants. In Canada basic food products were exempt from GST and sales tax was levied on carbonated beverages, confectionary and snack foods.

 Targeted agriculture subsidy programs had been operating for many years in the United States. Some countries included nutrition as part of social security approaches, but had only recently tended to focus on promoting healthier eating. Examples include the Women Infants Children program in the United States, and subsidisation of healthy school lunches, and of schools and local councils to provide fruit and vegetable vouchers to low income groups in England, and to pregnant women receiving benefits in Scotland. Very targeted initiatives were in place in some countries, such as the provision of fortified flour to Bedouin groups in Israel.

####  Restrict Food Advertising and Other Forms of Commercial Promotion (R)

 While around half the included policies (53%) mentioned restriction of advertising of unhealthy foods, most actions were expressed in formative terms. Six countries (32%) had introduced codes of ethics or voluntary frameworks for advertisers and the food industry; of these, two stated that they were monitoring progress and would consider regulation if voluntary action was too slow. Only three countries (England, Denmark and Israel) applied mandatory restrictions in some form. Some, for example Ireland and Nordic countries, reported seeking the introduction of broader controls (through the EU, for example) before acting.

####  Improve Nutritional Quality of the Whole Food Supply (I)

 Reformulation of unhealthy products was underway in most countries, as reported in 68% of the included nutrition policies. Most effort in this area was focused on product reformulation to reduce salt, with the aim of reducing blood pressure and cardiovascular disease. Other targets included replacing saturated/trans fats with healthier oils (eg, in the Spanish and EU policies) and reducing added sugars, especially sugar sweetened beverages (eg, England). The most common strategy was stated ‘consultation with’ or establishment of voluntary agreements with sections of the food industry (food manufacturers, suppliers and representative organisations). Three countries (England, Spain and the United States) also introduced approaches to restrict portion sizes, but several other documents only noted the need to address consumer confusion regarding serve sizes in nutrition labelling.

####  Set Incentives and Rules to Create Healthy Retail and Food Service Environments (S)

 Actions to create healthy retail and food service environments that support healthy eating were included in only four documents. Among the reported initiatives, England’s national policy included the most notable example of working with local authorities to improve supportive food retail environments, encouraging the use of planning powers to control the number and location of fast food outlets, particularly in proximity to schools. Similar approaches were underway in the United States, where zoning laws were proposed to discourage high availability of unhealthy food outlets around schools and also increase access to grocery stores and farmers markets in underserved neighbourhoods. France and Belgium had mandated provision of drinking water in public places. No policy actions specifically regarding commercial in-store retail promotions or restrictions were identified in this domain.

####  Policy Actions in the Food System domain (H)

The single policy area in the framework’s ‘Food system’ domain (H) is focused on harnessing the food supply chain and actions across sectors to ensure coherence with health (Figure 1, Table 2). Few actions in this domain were identified in the included policies, and there was little mention of the need to take a broad food systems approach to improve nutritional health. Local food supply projects reported in 6 (32%) of the reviewed policies included promotion of short food supply chains and farmers markets, as throughout Italy. Programs to promote local supply of fruit, vegetable and fish also operated across France, including subsidies for local retailers. The Irish, New Zealand and EU policies (16%) specifically included Health Impact Assessments as a tool to foster supportive social and physical food environments.

 There was no mention of the need to benchmark, assess and/or monitor various aspects of food systems, such as policy and/or food environments.

###  Policy Actions in the Behaviour Change Communication Domain

All of the included policy documents detailed actions around dissemination of information, education and improving food literacy and skills in the three areas of the NOURISHING framework’s ‘Behaviour change communication’ domain (ING) (Figure 1, Table 2).

####  Inform People About Food and Nutrition Through Public Awareness (I)

 All 19 nutrition policies (100%) mentioned public education to increase nutrition knowledge, but less than a quarter of these (21%) included information about unhealthy foods and drinks. Comprehensive, contemporary social marketing approaches were not common in the identified nutrition policies. One-fifth of countries reported conducting whole-of-population marketing programs focused on general nutrition, and a further 17% focused on increasing consumption of fruit and vegetables. Only 4 (21%) conducted social marketing campaigns targeting obesity; most of these had a focus on increasing physical activity, although some included a focus on fruit and vegetable intake also. The nutrition policies of England, Scotland, France and most Nordic countries included whole-of-population social marketing campaigns to reduce salt intake, Demark discouraged sweetened beverage consumption in children, and Italy’s plan discouraged ‘fast foods.’ Otherwise, no countries/regions promoted any ‘eat less’ messages. Promotion of optimal infant nutrition, reportedly conducted by 30% of included countries/regions, appeared to be more targeted, with less paid mass media as part of the marketing mix.

####  Nutrition Advice and Counselling in Healthcare Settings (N)

 All documents mentioned nutrition advice in healthcare settings. Countries with high prevalence rates of obesity, such as New Zealand, specifically included early intervention approaches in healthcare and community settings. About one in five (17%) policies noted the need for nutrition and public health education in undergraduate and post-graduate courses for a range of professions, such as nursing, medicine, the food industry and agriculture. A focus on workplace settings was more evident in recent policies, with 35% of included countries/regions offering programs in this area. Around two-thirds (65%) of countries reported actions to support professional development; while still a minority, the more recent policies were more likely to have extended professional development from the traditional hospital/community health/education sectors to the fitness (32%), catering (26%) and food industry (21%) sectors. No countries committed to building the size/capacity of the nutrition workforce.

####  Give Nutrition Education and Skills (G)

 Among interventions aimed at increasing demand for healthy foods, all country/regional policies (100%) included nutrition education for school children. Actions in some of the more recent policies were focused on increasing skills, such as cooking (eg, in England), rather than simply providing information, however such programs were relatively uncommon in the included policies. Nutrition education interventions targeting adults also were included in all national/regional policies, but tended to be centred on distribution of printed nutrition promotion resources. Again, the more recently published policies were more likely to include innovative and personalised delivery modes, through interactive websites for example, as identified in the policies of England, France and the United States. However, as at 2013, no policy mentioned the use of mobile communication channels such as ‘apps.’ More intensive targeted interventions were rarely mentioned, the exceptions being France, which provided telephone support for breastfeeding mothers, and Scotland, which referred to community-based lifestyle education groups. Only the Israel and Nordic region policies specifically noted the need to include a focus on reducing intake of unhealthy foods and drinks.

###  Summary of Policy Content

Figure 3 illustrates the proportion of the included policy documents that contained nutrition policy actions in each of the 10 areas of the NOURISHING framework.

Figure 3 illustrates clearly the dominant focus of policy documents on actions related to behaviour change communication (ING), followed by the provision of healthy foods in public institutions and other settings (O), then by another form of consumer education – food labelling (N), then reformulation (I). The relative lack of policy actions to tackle ‘unhealthy’ foods and drinks was clear (Figure 3).

###  Nutrition Policy Actions Not Captured in the NOURISHING Framework

Analysis also identified a wide range of policy actions in the included national/regional nutrition policies that did not align clearly with the domains and areas of the NOURISHING framework (Table 3). Among other areas, these related to: food safety, body dysmorphia and eating disorders, breastfeeding, population monitoring and surveillance, and environmental sustainability. For example, food safety was addressed in 26% of the included nutrition policies, and was a primary goal for the Czech Republic and a very strong focus for Japan.

 Regulation of the weight-loss industry was a specific focus in Italy, New Zealand and France. In France ethical standards also had been implemented around depiction of the “slim ideal” in magazines. One EU policy included a campaign aimed at reducing the social pressure to promote extreme thinness as a criterion of beauty, particularly among children and adolescents.

 While all policies included a focus on children, some also included specific actions to improve nutrition in other vulnerable groups, commonly infants and women, and also the elderly (eg, Denmark and Japan). In addition to Australia, Canada and New Zealand identifying the need to address diet-related health inequalities of Indigenous peoples as a priority, Canada’s national strategy also acknowledged the need for greater and sustained consultation with First Nations Peoples.

Few jurisdictions considered the two way relationship between environmental sustainability and diet. Six (32%) identified environmental sustainability as a key principle of their nutrition policy: Australia, England, New Zealand, France, Switzerland and the EU (Table 3). The focus generally was on partnership processes rather than outcomes, such as a commitment to working with other sectors, including agriculture. There was no evidence of any concerted action to address the impact of food choice on environmental sustainability up to 2013.

 Population-level food and nutrition monitoring and surveillance systems were identified for most countries/regions (87%), with 70% reporting regular data collection and reporting at least every five years. Among the most comprehensive examples were the reporting frameworks described in the national policies of the United States, France, Hungary and New Zealand. All included nutrition policies included monitoring and surveillance of diet-related risk factors and conditions; fewer (83%) reported regularly assessing food and nutrient intake.

The type of data collected at national levels appeared to vary widely, but data extraction was challenging as methods were not well described within policy documents. However, several key indicators of achievements, such as in Canada, centred on self-reported responses to short questions rather than much more robust and reliable dietary assessment methods.^56^ Similarly, not all countries aimed to physically measure height and weight anthropometrically to estimate body mass index and track obesity rates. Some of the more recently-published policies reported investment in capacity to conduct surveys and analyse data; England had established an Obesity Observatory and Spain planned to, while France was creating a Food Quality Observatory to monitor nutritional aspects of food products across the whole food system.

###  Other Elements of Comprehensive and Effective National Nutrition Policy

Several of the policies reviewed also included foundation tools and instruments and detailed governance mechanisms (process and structural factors) fundamental to broader system intervention (Table 3). Most Nordic countries, France, Hungary, Israel, Scotland and the United States presented measurable diet-related targets and goals. Most (68%) nutrition policies stated that they were based on relevant dietary guidelines, but less than half of these referred to dietary guidelines in setting specific aims, objectives or targets. About one-third (35%) of policy documents noted intent to report against targets, but some, including Australia and England, stated only that specific targets would be developed. Canada’s only dietary target was self-reported fruit and vegetable intake. Conversely, several other policies included targets, but did not mention intent to report against these. Intent to review and evaluate nutrition policy was reported in about two-thirds (65%) of the included policy documents. However, less than half of those included details of proposed approach; countries that referred to evaluation frameworks included France, Nordic countries, New Zealand, Scotland, and the United States. Countries that had reviewed their earlier policies, such as Denmark and France, tended to include more comprehensive indicators and more developed evaluation frameworks, subsequently.

Only England and Scotland reported details of funding in their nutrition policy documents. Similarly, mechanisms of development, consultation processes, governance and partnerships were not always clear. Whole-of-government, multi-sector approaches are acknowledged as being more effective in achieving nutrition outcomes,^5,57^ and the most recent policies adopted such an approach, with England’s a high profile example.

 Included policies demonstrated that nations were engaging a range of stakeholders in the policy process. However several countries/regions, including England, Ireland and the EU, reported involving ‘conflicted’ stakeholders with vested, commercial interests only in the implementation process rather than at the policy development stage.

## Discussion

###  Nutrition Policy Actions in 2013

Analysis of the available national nutrition policies of high-income countries in 2013 highlights an inadequate approach to intervening in food systems to deliver healthy diets and support environmental sustainability. The limited range, depth and breadth of the conventional nutrition policy actions identified, particularly the strong skew towards conservative behaviour change communication evident in all policies, would do little to help transform food systems or tackle food environments that encourage unhealthy eating by exploiting biological, psychological, social and economic vulnerabilities that reinforce preferences and demands for unhealthy choices.^1,5,8,58-60^

Across all NOURISHING domains there was a dominance of policy actions to promote consumption of ‘healthy’ food and drinks over those that aimed to restrict promotion or availability of ‘unhealthy’ options, including in the behaviour change communication domain (ING) (Figure 3). Few policy actions to decrease consumption of any foods or drinks (or packaging) were identified. In areas of the food environment domain (NOURIS), the most common policy actions were food labelling – also information-oriented – and provision of healthy foods in public institutions, mainly schools. Voluntary product reformulation was also relatively popular. There was little comprehensive policy action in the area of food systems; only half of the policies included intent to intervene in the food system specifically, and the majority of these inclusions were local level actions (H). Regulatory and legislative reforms were rarely included, despite being potentially the most cost-effective approaches.^61,62^ This is concerning in light of growing evidence that targeting food environments particularly through regulatory actions can have strong, long-term impacts on the health of populations.^61,62^

This over-reliance on education-based strategies, especially those promoting increased consumption, is consistent with a neoliberal approach, seen globally, that individualises responsibility for nutrition-related issues and promotes limited government intervention in regulating free enterprise.^63,64^ Previous studies have demonstrated that countries governed by parties with neoliberal ideologies often have low levels of political commitment for comprehensive, evidence-based strategies to decreasing poor nutrition in the population.^63-65^ If high levels of political commitment are not present in a country this can result in result in tokenistic policies with limited resources, often designed to appease powerful interest groups.^5,7,10,58,64,66^ In this study, national nutrition policies tended to be more comprehensive if conflicted stakeholders, with vested commercial interests, were involved only in the implementation stage (rather than at the policy development phase), as was seen in England, Ireland and the EU. This approach is now recommended by the WHO.^66^ Some countries with a strong food culture, such as Italy and France, proposed memorandums of understanding with sectors beyond health to foster action to improve nutrition determinants, and developed relatively comprehensive food-focused nutrition policies that included multiple actions to improve sustainability of local food systems. Details of coordination mechanisms were more likely to be provided in policy documents that addressed other risk factors in addition to nutrition, including Canada and the United States. However, these documents also provided fewer details of potential policy actions and were more formative in approach to nutrition policy actions specifically. Finally, very few of the policies in this study clearly identified any resourcing or capacity to support implementation. This omission has been identified previously in the literature on the political economy of nutrition as an impediment to implementing nutrition actions.^67,68^ These observations support the need to ensure transparency, rigour and public scrutiny of government food and nutrition policy, regulatory and norm-setting activities to ensure they are adequately protected from undue commercial interest.^66^

###  International Recommendations for Nutrition Policy Development and Recent Progress

Recommendations by the WHO^9,57,66^and other authoritative organisations such as the Nutrition and Obesity Policy Research and Evaluation Network^69^ are now used more actively to guide development to ensure that nutrition policies are comprehensive and multi-sectoral. To be comprehensive and effective, nutrition policies must reflect clear goals and targets, and be underpinned by foundational tools, including country-specific food-based dietary guidelines, nutrient reference values, and population monitoring and surveillance systems. It is also imperative that process and structural factors are considered, such as best-practice, transparent governance structures and coordination mechanisms, policy integration, adequate, dedicated funding for implementation, and performance monitoring, evaluation and review.^9,57,70^ In this study, policies that reflected these recommendations^9,57,69^tended to be more comprehensive (Table 2).

Some deficiencies of national/regional nutrition policies are now being addressed. A recent study comparing aggregate implementation scores for 151 countries based on their implementation of 18 WHO-recommended policies to address NCDs from 2015 to 2017^71^ found improvements in the areas of nutrition as well as tobacco, but reductions in alcohol and physical activity. However, that study was positioned at a relatively high level, with little granularity to support detailed comparison of the depth and breadth of specific nutrition policy actions provided in this review. For example, this current review found that no countries up until 2013 were benchmarking, assessing and/or monitoring key aspects of the food and nutrition system such as food environments or policy action at the national level. While food environment metrics were being investigated in some countries at the time of the study, the urgent work of developing and implementing standardised methods for data collection and analysis is now being progressed globally by the International Network for Food and Obesity/Non-communicable Diseases Research, Monitoring and Action Support (INFORMAS).^59^

The study was limited to OECD members, as the original intent was to review policy activities in countries with similar politico-socio-economic systems to Australia and at the time it was considered that these countries would be more likely than low and middle income countries to invest resources into the development of nutrition policy actions.^16^ However, since 2013, while there has been a dramatic rise in activity in some OECD countries, particularly Mexico,^72,73^ greater trajectory in nutrition policy action has been observed in several low and middle income countries,^60,71^ with Chile (now a high-income country^1^) a high profile example.^5,74^ For instance, while the red colour in ‘traffic light’ food labels may have been perceived as a warning by some consumers previously, in 2016 Chile introduced specific, bold warning labels and strict food marketing regulation, which evaluations suggest have been more effective.^75,76^ Policy action in low and middle income countries may indicate a preference for prevention over reliance on expensive clinical treatments for diet-related diseases, as observed in many high-income countries.^77^ Importantly, nutrition policy action in low and middle income countries has occurred often against a backdrop of immense food industry pressure against any policy change.^78-80^

The WHO reviewed country progress in nutrition policy in 2009-2010^9^ and 2016-2017,^60^ reporting self-reported regional progress toward achieving several targets and commitments globally.^60^ Progress was reported in six key action areas related to: infant and young child nutrition; school health and nutrition programmes; promotion of healthy diet and prevention of obesity and diet-related NCDs; vitamin and mineral nutrition; prevention and treatment of acute malnutrition; and nutrition and infectious disease. Although only included in 37% of nutrition policies until 2013, the WHO found that breastfeeding counselling was the intervention most often reported to be implemented in all countries in all regions in 2017. The accuracy of self-reported activities is unknown, and the data provided generally lacked the granularity commonly reported by countries using the NOURISHING framework. However, the more detailed results of this systematic scoping review could provide a retrospective baseline, and the methods could be replicated to compare content, scope and range of nutrition policy actions over time in OECD countries.

###  Nutrition Policy Actions Not Captured in the NOURISHING Framework

When the original scoping study was conducted,^16^ there was not an accepted international nutrition policy framework to categorise nutrition policy actions, so a bespoke framework specific to the Australian context, based on the content of (non-conflicted) stakeholder submissions to relevant government strategies and plans, was developed.^16^ A number of global policy frameworks, of different scope, have been developed subsequently to classify potential nutrition actions.^5,11^ Some are relevant to specific countries or issues, for example, Food-PRICE in the United States,^81^ others focus on agriculture,^82,83^ while those encompassing broader food systems tend to be set descriptively at a relatively high level rather than identifying leverage points for potential intervention.^2,3,58^ The NOURISHING framework was applied in this study as it is practical and popular; is applicable internationally, yet its focus on obesity and NCDs is highly relevant to high-income economies; provides flexibility to shape local responses; fosters categorisation, reporting and monitoring; and is accompanied by a frequently updated database that provides a global overview of implemented government policy actions.^5,11,14^ Although the list of policy actions captured in the NOURISHING Framework is sizeable, it has been clarified recently on the WCRF website that this should not be taken as exhaustive.^11,13^ This study suggests that the NOURISHING framework could be expanded and strengthened to better encompass the health and sustainability dimensions of food systems in a number of areas. Firstly, by the inclusion of detailed policy actions focused on interventions to promote environmental sustainability in the food systems domain, including those to help reduce carbon footprint, water use, fertiliser use and increase biodiversity.^1-3^ This could be informed by more recent work that has described the global syndemic of obesity, undernutrition and climate change^1^ and the actions needed to build healthier food systems through understanding the broader “food-health nexus,”^84^ including actions to reduce waste, excess energy intake, intake of unhealthy foods and drinks and healthy plant-based food choices.^1-3^

Additionally, the Food system domain (H) could be extended also to include more detailed governance mechanisms and program management principles.^27,57,85^ The specific inclusion of targeted policies to improve nutrition in vulnerable groups, such as breastfeeding,^1,86^ would strengthen equity. However, given the current focus on obesity and NCDs, some other frequently mentioned nutrition policy actions in the area of food safety, body dysmorphia and disordered eating, do not fit so readily in the framework.

While the NOURISHING framework now includes development of food-based dietary guidelines (although only under the area ‘I’ – informing the public – whereas these have broader policy and practice applications),^13^ the framework also could be expanded to include ongoing commitment to the development of other foundation tools, such as country-specific nutrient reference values and regular, comprehensive food and nutrition monitoring and surveillance systems. Ideally, the latter would be expanded to assess, benchmark, survey and report key food system components such as nutrition knowledge and attitudes, food supply, food costs and other aspects of choice architecture and the food environment, as identified by INFORMAS.^59^ With these modifications, the resulting framework could be sufficiently broad to accommodate policy actions to address malnutrition in all its forms^87^ and support a more transformational food systems approach.^2,3,5,88^

###  Limitations

The search strategy to identify national nutrition policies appeared to be robust as it identified the existence of over 95% of the in-scope countries, key multi-country and regional nutrition policies included in the concomitant scans of the draft report in 2010 of the WHO’s review of nutrition policies.^89,90^ It is possible that some countries may have developed nutrition policies that were not available in the literature or on websites. However, it was considered that policies that were not publicly accessible were unlikely to be implemented. Sub-country level policies were excluded and this resulted in lack of consideration of some innovative nutrition policy approaches, such as those attempted and/or applied in the states of California^91^ and New York^92^ in the United States, and Queensland in Australia.^93^ Most of the nutrition policies included had a strong foundation in the health sector; given different terminology used by different sectors, other relevant ‘stand-alone’ initiatives, such as the European Fund for the Most Deprived^94^ or exemption of GST from basic, healthy foods in Australia outlined in Treasury documents^95^ may have been missed. Similarly, policy actions to address nutrition priorities emerging in 2013, such as palm oil and other specific environmental sustainability issues, may also have been missed due to the long lead time around formal policy development. Restricting the search to policies published in English also limited the results; however, OECD member nations usually produce English versions of key documents.

 Classification of some specific policy actions against the domains of the NOURISHING framework may have been arbitrary and include potential overlap, as is likely to be the case with application of other nutrition policy frameworks.

The original systematic review was completed in 2013 and documents were identified and accessed online. Therefore, some policy documents were no longer at the URL where they were previously located and current URLs are provided in Table 1. However, only two documents (Belgium’s and Denmark’s superseded policy) could no longer be located.

As the documents were written at different levels, it was difficult to define the scope of a ‘policy action’ and therefore to enumerate specific policy actions for each nation/region. Hence Table 2 captures any inclusion of relevant policy action in each domain and policy area of the NOURISHING framework, with more detail included in the text. A specific taxonomy of nutrition policy action could be developed to aid future analysis. Further, the lack of standard, global definitions of ‘healthy’ and ‘unhealthy’ food and drinks potentially affects intent, interpretation and classification of relevant policy actions. Further, most nutrition policies reviewed in 2013 implied assessment of the ‘healthiness’ of foods on the basis of nutrient-profiling – a reductionist approach that can penalise some traditional, healthy foods, particularly on the basis of their fat content.^96^

This study did not report evaluations of the included nutrition policies, which could be considered a limitation. The few evaluations of nutrition policies that were available in 2013 are reviewed in the scoping study.^16^ Lack of specific goals and targets made evaluation of nutrition policies challenging. Without evaluations, it is not clear whether policy actions were implemented at all or as intended, of if they had the desired impact or contributed to outcomes. When assessing effectiveness of nutrition policies, the scoping study found that bias in the types of policy actions implemented globally, and the paucity of systematic, objective process, impact, outcome and economic evaluations were highly problematic.^16^

## Conclusion

 Analysis of jurisdictional nutrition policies of OECD countries as at 2013 has provided valuable insights and could be used as a retrospective baseline for future assessment, monitoring and surveillance of policy action. The NOURISHING framework could be expanded to provide an even more comprehensive nutrition policy framework, especially with inclusion of additional nutrition policy actions to promote environmental sustainability, good governance, and equity.

This study found that only seven years ago, half of the most developed economies globally did not have a publicly available nutrition policy. Further, among countries that did, there was a strong focus on nutrition education strategies, consistent with a neoliberal approach centring individual responsibility. Little government intervention targeted commercial enterprise; voluntary processes were favoured and regulatory and legislative reforms were rarely included. There was relatively little policy action targeting over-consumption of ‘unhealthy’ food or drinks. Those nutrition policies developed in tandem with stakeholders with vested commercial interests tended to be less comprehensive. No examples of the necessary multi-strategy, inter-sectoral, coordinated, evidence-based policies required to drive systemic change were identified. Therefore, it is not surprising that rates of obesity and diet-related conditions have continued to rise in these jurisdictions, nor that governments are currently off-track to deliver the systemic transformation required to meet global nutrition, NCD and sustainable development goals.^1,8,60^

## Acknowledgements:

The search strategy for the original, broader systematic literature review comprising the scoping study to inform a new nutrition policy in Australia^[1]^ was developed in 2013 with Dr. Alison Weightman and Dr. Helen Morgan of the Support Unit for Research Evidence at Cardiff University, Wales, the United Kingdom, and Professor Philip Baker of the Queensland University of Technology. The SURE team, including Dr. Fiona Morgan, ran the searches of databases for peer-reviewed literature, de-duplicated outputs and supplied a full set of results as EndNote libraries. Dr. Vicki James searched national websites, downloaded relevant grey literature and extracted data for the relevant research question in the scoping study^[1]^.Thank you to the co-authors of the original systematic literature reviews, Dr. Rosemary Stanton, Professor Sharon Friel and Professor Kerin O’Dea, who assisted with interpretation of the evidence identified in the scoping study.

## Ethical issues

 Not applicable.

## Competing interests

 Authors declare that they have no competing interests.

## Authors’ contributions

 All authors attest they meet the ICMJE criteria for authorship. AL conceptualised, designed and supervised the study, and acquired the data. All authors contributed to analysis and interpretation of data and development of the manuscript. AL critically revised the manuscript. All authors reviewed and approved the final manuscript.

## Funding

No funding was provided for this project. The original literature review comprised part of the 2013 Scoping Study to inform the development of the new National Nutrition Policy^[1]^ conducted by the Queensland University of Technology, funded by the Australian Government Department of Health and Ageing in response to request for tender RFT 028/1213.

## Endnotes


^[1]^The World Bank reclassified Chile from an Upper Middle to a High Income country in 2013: https://blogs.worldbank.org/opendata/new-country-classifications.


## Supplementary files

Supplementary file 1. Detailed Search Strategy Including Full Electronic Search Strategy for at Least One Database.
