# Supplementary material for: Achieving Food System Transformation: Insights From A Retrospective Review of Nutrition Policy (In)Action in High-Income Countries
Source: Int J Health Policy Manag. 2020 Oct 19;10(12):766–83. doi: 10.34172/ijhpm.2020.188 (PMC9309976; doi:10.34172/ijhpm.2020.188)
Supplement: Supplementary file 1 — Detailed Search Strategy Including Full Electronic Search Strategy for at Least One Database. [file ijhpm-10-766-s001.pdf]

Supplementary file 1. Detailed Search Strategy Including Full Electronic Search Strategy for at Least One Database.

This supplementary document details the search strategy for a systematic review conducted as part of a comprehensive scoping study [1], the results of which were used to reclassify and analyse nutrition policy actions against the World Cancer Research Fund's NOURISHING framework [2].

Search for national food and nutrition policies (in scope countries)

Systematic review methods

The project team aimed to use methods which were systematic, transparent and potentially replicable. The process was based upon the principles of robust systematic summary outlined in the Cochrane Handbook for Systematic Reviews [3] and pragmatically tailored for this project as detailed in the report of the scoping study [1]. Each team member was trained in the process and quality governance measures were implemented.

The overarching process is summarised in Figure 1.

Figure 1 Overarching process

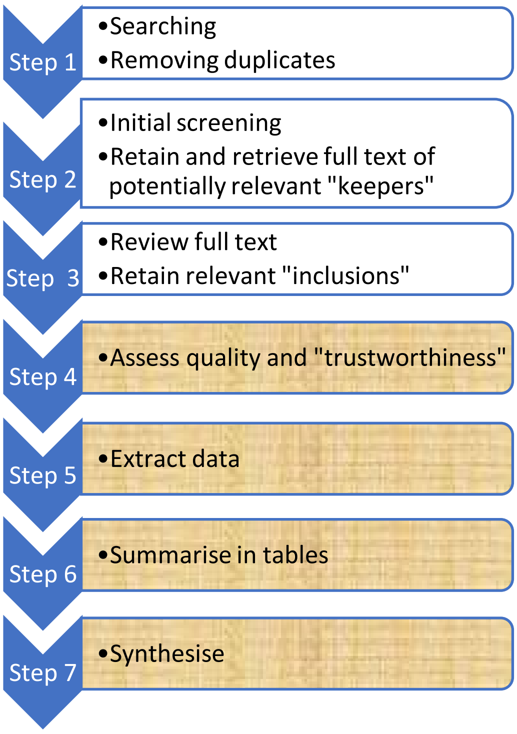

Development of search strategy

The search strategy was developed by Dr Alison Weightman and Dr Helen Morgan of the Support Unit for Research Evidence (SURE) at Cardiff University, Wales, UK and Professor Philip Baker and Professor Amanda Lee of the Queensland University of Technology (QUT) using standard methods [4,5].

Development of the search strategy involved:

- Articulation of searchable question/s and sub-questions, and development of the PICO-T components of each research question (see below).
- Identification and definition of search terms;
- Identification of potential databases, websites and receipt of proffered documents by the Department of Health and Ageing (DoHA) for consideration for inclusion.
- Testing of the search strategy in Medline (Ovid), which was then adapted to other databases.
- Approval of the literature search strategy by the DoHA (22 March 2013).

A series of searchable, answerable questions were derived from the main, over-arching research questions before designing the review, as *post-hoc* questions are more susceptible to bias than those determined *a priori* [6].

Each searchable, answerable question followed the PICO–T concept. The acronym translates to:

P – Populations/People/Patients/Problem
I – Interventions(s)/Issues
C – Comparison
O – Outcome
and additionally
T – Type of study design appropriate for the question

After developing the PICO – T structure, each question was also restated in 'plain language'.

Other than where identified as being conducted by the QUT team, the SURE team ran the searches, de-duplicated outputs and supplied a full set of results as EndNote libraries or table format as a word document to the QUT team. For each research question the QUT team developed screening tools, quality assessment tools and data extraction tools. These are included in the scoping study report (sections A2.2.5).

For each research question, the QUT team screened the titles and abstracts of all returns, identified documents of potential relevance, retrieved full versions of these papers and reports and conducted a second screen to identify relevant documents, included these in final Endnote libraries and completed PRISMA flow diagrams.

For each research question data was then extracted from the included documents and entered into data extraction tables.

Overview of Research Question

Plain language question: Which *in scope* (OECD) countries have food and nutrition polices?

Sub- questions:

a) What are the scope, issues, recommendations, inclusions and format of these (quality) food and nutrition policies in OECD countries?

b) What approaches and information have been used to determine the scope, issues, recommendations, inclusions and format of these food and nutrition policies?

Detailed search strategy

Search for: Existence of any OECD countries' (national) food and nutrition policies and component strategies (identifying the most current)

Study type: Food and Nutrition: policies, policy, strategy, plan, report, monitoring, surveillance, guideline, guidance, legislation, program, project, intervention, initiative, regulation, law, directive, evaluation.

Identification and definition of search terms:

Search terms for each PICO–T element were identified through reference to the simple logic model in accordance with best-practice for systematic reviews [7]. The search terms were subsequently refined by the SURE expert information specialists and trialled on Medline biographic dataset as standard practice with the application of the appropriate Boolean operators to expand (or) and join (and) the elements. A specialty OECD-countries filter was integrated to exclude non-OECD countries. The search strategy was then adapted to all specified databases.

For each question, the search strategies were initially constructed, trialled, discussed, refined and then agreed upon by the DoHA. All aspects of the searching process were documented to ensure transparency and reproducibility.

Databases and websites searched

The choice of databases was tailored to the questions and the selection extended beyond the area of health and included other relevant areas such as agriculture, environment and transport.
Databases searched included:

- Cochrane Public Health Group Specialised Register
- The Cochrane Library
- MEDLINE, MEDLINE In-Process
- EMBASE
- CINAHL
- PsycINFO
- LILACS
- ASSIA
- British Nursing Index
- EPPI Centre
    - DoPHER
    - TRoPHI
- ERIC
- Sociological Abstracts
- Transport Database TRIS
- Web of Science:
    - Science Citation Index, Social Sciences Citation Index and Conference Proceedings Citation Index
- Agricola
- Agris
- AANRO (Australian agriculture and natural resources online)
- HEED
- NEED

Websites searched included:

- EU Platform on Diet, Physical Activity and Health http://ec.europa.eu/health/ph_determinants/life_style/nutrition/platform/database/
- Health-evidence.ca (now Health Evidence™)
    - http://health-evidence.ca (now www.healthevidence.org)
- International Union for Health Promotion and Education
    - http://www.iuhpe.org
- Health Technology and Assessment Programme
    - http://www.ncchta.org
- NICE guidelines
    - http://www.nice.org.uk
- SIGN guidelines
    - http://www.sign.ac.uk
- US Centres for Disease Control and Prevention
    - http://www.cdc.gov/
- World Health Organization
    - http://www.who.int/en/
- Food and Agricultural Organisation
- Country-specific (international) government websites.

Time: 2002 to 2013. Literature was searched for the previous 12 years to ensure that the policies and evaluations located reflect contemporary issues and practice such as prevention of chronic disease and obesity. For example, several countries had nutrition strategies that focused on ameliorating nutrient deficiencies and under nutrition before then. It is also important to ensure that identification of multiple duplicates and superseded policies in some countries is minimised. Where an identified country may have more than one relevant policy during this period, the most recent was assessed.

Population:

- In scope countries defined as those with similar socio-economic/political conditions as Australia and members of the OECD.

Australia	France	Korea	Slovenia
Austria	Germany	Luxembourg	Spain
Belgium	Greece	Mexico	Sweden
Canada	Hungary	Netherlands	Switzerland
Chile	Iceland	New Zealand	Turkey
Czech Republic	Ireland	Norway	United Kingdom
Denmark	Israël	Poland	United States
Estonia	Italy	Portugal	
Finland	Japan	Slovak Republic	

- **Inclusions:**

- Policies targeting all component population groups in these countries.

- **Exclusions:**
  - Policies targeting those with serious medical conditions; elite athletes; frail elderly in institutions
- **Intervention:** Development of food and nutrition policy
- **Comparator:** Lack of food and nutrition policy
- **Outcomes:**
  - The existence of country food and nutrition policies and policy actions that sit under this policy framework.

Sample search strategy and returns

The sample search strategy is included below in Table 1.

Table 1 Sample search strategy

Database(s): Ovid MEDLINE(R) without Revisions 1996 to February Week 4 2013 Search Strategy:

#	Searches	Results
1	((food or beverage* or drink* or water or nutrition* or nutrient* or diet* or obesity or agricultur* or land-use) adj3 (policies or policy or strateg* or plan* or report* or surveillance or guideline* or guidance or legislation or program* or intervention* or initiative* or regulat* or law* or directive*) adj5 (nation* or government* or country* or federal)).ti,ab.	959
2	Nutrition Policy/	5283
3	Food Supply/	4494
4	Food Assistance/	16
5	exp Food Handling/	21766
6	food packaging/ or food labeling/	3895
7	exp food quality/	38306
8	Legislation, Food/	1282
9	exp Agriculture/	40603
10	exp beverages/ or exp food/	524230
11	Food Services/	2264
12	dietetics/	2316
13	exp Diet/	94688
14	Obesity/	77197
15	or/3-14	686655

16	((policies or policy or strateg* or plan* or report* or surveillance or guideline* or guidance or legislation or program* or intervention* or initiative* or regulat* or law* or directive*) adj5 (nation* or government* or country* or federal)).ti,ab.	57596
17	2 and 15 and 16	348
18	1 or 17	1167
19	(Algeria$ or Egypt$ or Liby$ or Morocc$ or Tunisia$ or Western Sahara$ or Angola$ or Benin$ or Botswana$ or Burkina Faso or Burundi$ or Cameroon or Cape Verde or Central African Republic or Chad$ or Comoros or Congo or Djibouti or Eritrea$ or Ethiopia$ or Gabon$ or Gambia$ or Ghana$ or Guinea or Keny$ or Lesotho or Liberia$ or Madagasca$ or Malawi$ or Mali or Mauritania or Mauritius or Mayotte or Mozambiq$ or Namibia$ or Niger or Nigeria$ or Reunion or Rwand$ or Saint Helena or Senegal$ or Seychelles or Sierra Leone or Somalia$ or Somali or South Africa$ or Sudan$ or Swaziland or Tanzania$ or Togo or Ugand$ or Zambia$ or Zimbabw$ or China or Chinese or Hong Kong or Macao or Mongolia$ or Taiwan$ or Tibet$ or Belarus or Moldov$ or Russia$ or Ukrain$ or Afghanistan or Afghani or Armenia$ or Azerbaijan$ or Bahrain$ or Cyprus or Cypriot or Georgia$ or Iran$ or Iraq$ or Jordan$ or Kazakhstan$ or Kuwait$ or Kyrgyzstan or Leban$ or Oman or Pakistan$ or Palestin$ or Question Atar or Saudi Arabia$ or Syria$ or Tajikistan or Turkmenistan or United Arab Emirates or Uzbekistan or Yemen or Bangladesh$ or Bhutan$ or British Indian Ocean Territory or Brunei Darussalam or Cambodia$ or India$ or Indonesia$ or Lao or People's Democratic Republic or Malaysia$ or Maldives or Myanmar or Nepal$ or Philippin$ or Singapore$ or Sri Lanka$ or Thai$ or Timor Leste or Vietnam$ or Albania$ or Andorra or Bosnia$ or Herzegovina$ or Bulgaria$ or Croatia$ or Faroe Islands or Greenland or Liechtenstein or Lithuani$ or Macedonia or Malta or Maltese or Romania$ or Serbia$ or Montenegr$ or Svalbard or Argentina$ or Belize or Bolivia$ or Brazil$ or Colombia$ or Costa Rica$ or Cuba$ or Ecuador$ or El Salvador$ or French Guiana$ or Guatemala$ or Guyana or Haiti$ or Honduras or Honduran or Jamaica$ or Nicaragua$ or Panama$ or Paraguay$ or Peru$ or Puerto Ric$ or Suriname or Uruguay$ or Venezuela$ or developing countr$).ti,sh.	454765
20	18 not 19	959
21	limit 20 to yr="2002 -Current"	761

Screening

The returns were screened in two stages to identify relevant National nutrition policies, strategies and related interventions. The screening tools are included in Box A and Box B below. The first screening tool was applied to returned titled and abstracts to identify all those returns that appeared to be national nutrition policies. The full documents of these “keepers” were retrieved by web searches, and the second screening tool was applied to full documents to identify all those “keepers” that were national nutrition policies.

The PRISMA flow diagram has been supplied separately.

Box A Initial screening tool

Initial Screening Tool Question Bii:

Aim: To identify documents that *appear to* be national level (food and) nutrition policies (KEEPERS)

Definitions:

Policy: written statement of commitment (generally written in broad terms) by a nation state. Strategy may be similar to a policy.

Action plans: (National plans of action on nutrition, NPANs) arise from policy, providing more detailed operational plans of action; include budgets and specific goals and targets that are specific, measurable, attainable, relevant and time bound (SMART).

Programme: provides the detail of the implementation of the action plan; with specific projects defined within

National: country level (Should only have country level returns for Bii)

Does the Title or Abstract state:

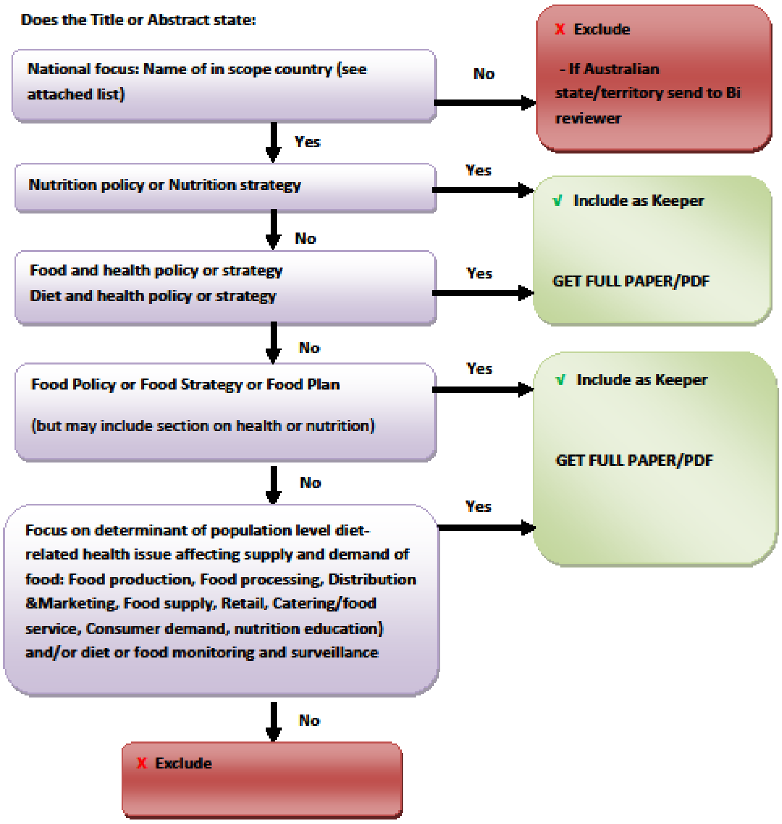

Box B Second screening tool

Second Stage Screening Tool Question Bii:

Aim: To identify national level (food and) nutrition policies

SEARCH OF FULL TEXT DOCUMENTS: Contents; Summaries; full text

Definitions:

<u>Policy:</u> written statement of commitment (generally written in broad terms) by a nation state. Strategy may be similar to a policy.

<u>Action plans:</u> (National plans of action on nutrition, NPANs) arise from policy, providing more detailed operational plans of action; include budgets and specific goals and targets that are specific, measurable, attainable, relevant and time bound (SMART).

<u>Programme:</u> provides the detail of the implementation of the action plan; with specific projects defined within

<u>National</u>: country level (Should only have country level returns for Bii)

Does the Contents, Summary, Full text document include:

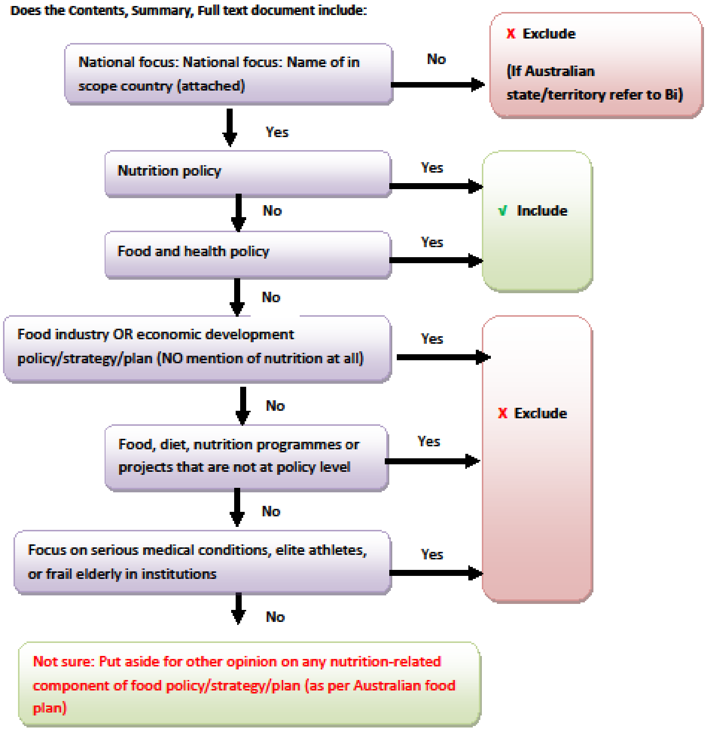

References

1. Lee A, Baker P, Stanton R, Friel S, O'Dea K, Weightman A. Scoping study to inform development of the National Nutrition Policy for Australia (report to the Australian Government Department of Health and Ageing). Released in response to a freedom of information (FOI) request, March 2016. Available at: http://webarchive.nla.gov.au/gov/20171113055450/http://www.health.gov.au/internet/main/publishing.nsf/Content/foi-074-1516 (accessed October 2019)2013.
2. World Cancer Research Fund. NOURISHING framework and database. https://www.wcrf.org/int/policy/nourishing/our-policy-framework-promote-healthy-diets-reduce-obesity. Accessed 14 October, 2019.
3. Higgins JPT, Green S, eds. *Cochrane Handbook for Systematic Reviews of Interventions Version 5.1.0 [updated March 2011]*: The Cochrane Collaboration; 2011.
4. Centers for Disease Control and Prevention (CDC). *Systematic Review Methods*: 2012.
5. National Health and Medical Research Council. *How to review the evidence: systematic identification and review of the scientific literature.* Canberra: Commonwealth of Australia; 2000.
6. Health Evidence. Developing an efficient search strategy using PICO. http://www.healthevidence.org/practice-tools.aspx#PT2. Accessed December, 2019.
7. Anderson L, Petticrew M, Rehfuess E, et al. Using logic models to capture complexity in systematic reviews. *Res. Synth. Methods.* 2011; 2(1):33-42. doi:10.1002/jrsm.32
